# Deficiency of 15-LOX-1 Induces Radioresistance through Downregulation of MacroH2A2 in Colorectal Cancer

**DOI:** 10.3390/cancers11111776

**Published:** 2019-11-11

**Authors:** Yoo Jin Na, Bo Ram Kim, Jung Lim Kim, Sanghee Kang, Yoon A. Jeong, Seong Hye Park, Min Jee Jo, Jeong-Yub Kim, Hong Jun Kim, Sang Cheul Oh, Dae-Hee Lee

**Affiliations:** 1 Graduate School of Medicine, Korea University College of Medicine, Seoul 02841, Korea; wing1278@naver.com (Y.J.N.); leomi2614@naver.com (Y.A.J.); psh3938@hanmail.net (S.H.P.); minjeeyoyo@nate.com (M.J.J.); 2 Department of Oncology, Korea University Guro Hospital, Korea University College of Medicine, Seoul 08308, Korea; ilovewish777@naver.com (B.R.K.); clickkjl@naver.com (J.L.K.); 3 Department of Surgery, Korea University Guro Hospital, Korea University College of Medicine, Seoul 08308, Korea; kasahal@korea.ac.kr; 4 Division of Radiation Cancer Research, Korea Institute of Radiological and Medical Sciences, Seoul 01812, Korea; wjdduql@hanmail.net; 5 Division of Oncology, Department of Internal Medicine, Kyung Hee University School of Medicine, Seoul 02447, Korea; xpassion84@naver.com; 6 Department of Marine Food Science and Technology, Gangneung-Wonju National University, 120 Gangneung, Gangwon 210-702, Korea

**Keywords:** 15-LOX-1, colorectal cancer, radiation, macroH2A2, DNA damage

## Abstract

Despite the importance of radiation therapy, there are few radiation-related markers available for use in clinical practice. A larger catalog of such biomarkers is required to help clinicians decide when radiotherapy should be replaced with a patient-specific treatment. Arachidonate 15-lipoxygenase (15-LOX-1) enzyme is involved in polyunsaturated fatty acid metabolism. When colorectal cancer (CRC) cells were exposed to radiation, 15-LOX-1 was upregulated. To verify whether 15-LOX-1 protects against or induces DNA damage, we irradiated sh15-LOX-1 stable cells. We found that low 15-LOX-1 is correlated with radioresistance in CRC cells. These data suggest that the presence of 15-LOX-1 can be used as a marker for radiation-induced DNA damage. Consistent with this observation, gene-set-enrichment analysis based on microarray experiments showed that UV_RESPONSE was decreased in sh15-LOX-1 cells compared to shCon cells. Moreover, we discovered that the expression of the histone H2A variant macroH2A2 was sevenfold lower in sh15-LOX-1 cells. Overall, our findings present mechanistic evidence that macroH2A2 is transcriptionally regulated by 15-LOX-1 and suppresses the DNA damage response in irradiated cells by delaying H2AX activation.

## 1. Introduction

Colorectal cancer (CRC) is the third most common cancer and the third leading cause of cancer-related deaths worldwide. Surgery is generally recommended for its treatment, but in practice, radiation therapy and chemotherapy are concurrently implemented. Radiation therapy is administered to more than half of all cancer patients worldwide to reduce the tumor size. It is especially important for CRC because it can be performed on the anus in situ and, thus, prevents or delays amputation of the anus. However, the problem with this therapeutic modality is that radiation effects vary dramatically among patients. Moreover, the effects of radiation therapy on radiation resistance are rarely studied [1,2,3,4].

The enzyme 15-LOX-1 is known to be the main metabolic enzyme for linoleic and arachidonic acids. The 13-S-hydroxyoctadecadienoic acid produced by 15-LOX-1 is the main transcription factor responsible for killing cancer cells [5,6]. The activity of 15-LOX-1 opposes [7,8,9] that of COX-2, which is responsible for radioresistance of cancer cells [10,11,12]. Accordingly, 15-LOX-1 was pursued in this study to find out its relationship with radiosensitivity. Recently, 15-LOX-1 was proposed to suppress hypoxia-inducing factor-1 and angiogenesis in several reports [13,14,15,16], which also suggests that 15-LOX-1 is a central factor in radiation sensitivity. However, to date, the relationship between 15-LOX-1 and radiation sensitivity has not been clarified.

DNA double-strand breaks (DSBs) generated by ionizing radiation (IR) can cause cell-cycle arrest, senescence, and even cell death. Once DSBs are generated in the DNA, its fate is decided by multiple regulators, such as sensors (MRN), transducers (ATM and Chk2), mediators (H2AX, 53BP1, MDC1, and BRCA1), and effectors (p53). The first step constitutes the recognition of DNA damage. In this step, the mammalian MRN complex (MRE11-RAD50-NBS1) recognizes DNA DSBs and recruits ATM serine/threonine kinase. DNA-binding of these proteins induces the γH2AX signal, which recruits the DDR (DNA damage response) proteins [17,18,19,20,21].

Here, we demonstrate that the radiation-induced upregulation of 15-LOX-1 increases IR-induced cell death and DNA damage as macroH2A2, which is transcriptionally regulated by 15-LOX-1, decreases the DNA damage response by delaying H2AX activation. These results suggest that modulation of 15-LOX-1 may help overcome tumor radiation resistance in patients and that 15-LOX-1 can be used as a patient-specific prognostic marker for radiation therapy.

## 2. Results

### 2.1. Radiosensitivity of CRC Cell Lines Correlates with 15-Lox-1 Expression Levels

**Figure 1:**
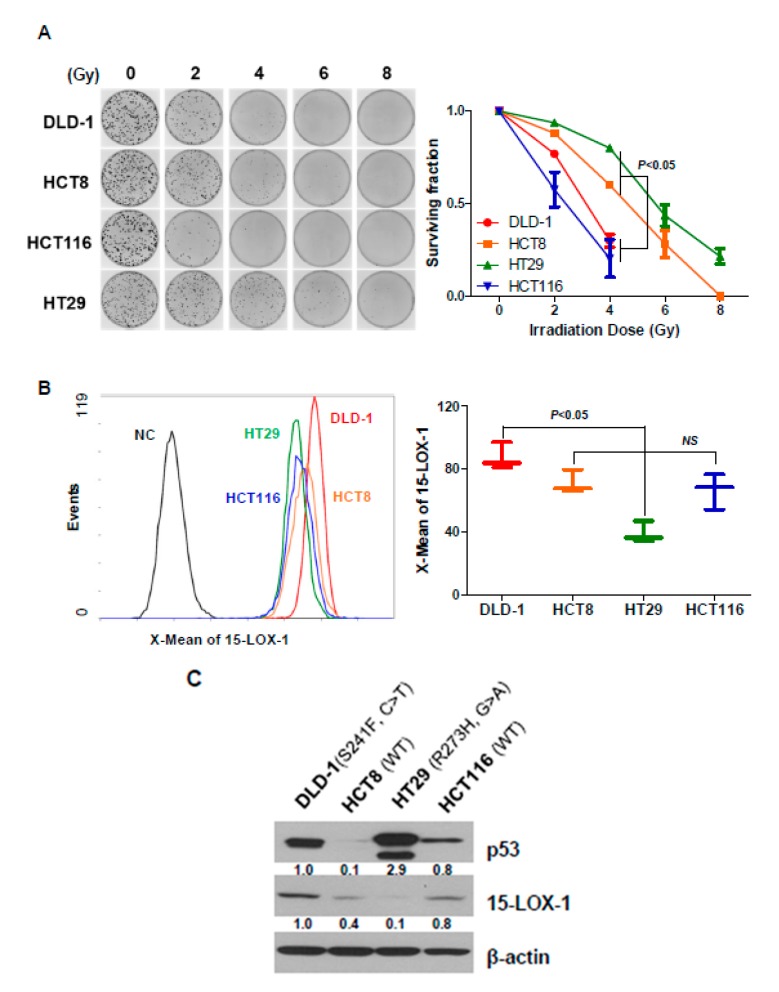
The radiosensitivity of CRC cell lines correlates with 15-LOX-1 expression levels. (**A**) Representative clones and clonogenic cell survival curves of DLD-1, HCT8, HCT-116, and HT29 cells. After seeding, the cells were irradiated at 2, 4, 6, and 8 Gy. The numbers of the colonies generated were counted two weeks later. (**B**) The 15-LOX-1 level of CRC cells was measured by flow cytometry. (**C**) The p53 and 15-LOX-1 levels were analyzed by Western blotting. The functional statuses of p53 in the cell lines are indicated above the results to describe their correlation with the role of 15-LOX-1 in radiation sensitivity.

Although the regulation of lipoxygenases, such as 15-LOX-2, 12-LO and 5-LO, has been shown in several reports to contribute to radiosensitivity [22,23,24], this study constitutes the first analysis of the relationship between 15-LOX-1 expression and radiation response in CRC cell lines. We measured the radiosensitivity of CRC cell lines by using clonogenic cell survival assays (Figure 1A). After 14 days of irradiating DLD-1, HCT8, HCT29, and HT116 cells (2, 4, 6, and 8 Gy), HT29 and HCT8 cells were found to be more resistant to radiation than DLD-1 and HCT116 cells. The differences among the groups were statistically significant at 4 Gy (*p* < 0.05). The levels of 15-LOX-1 protein were measured by flow cytometry to evaluate whether they correlated with radiosensitivity (Figure 1B). No statistically significant difference in 15-LOX-1 expression levels between the radiation-sensitive and -insensitive groups (described in Figure 1A) was identified.

The transcription factor p53 is known to control radiation sensitivity [25,26,27,28]. Except in a few reports, p53 dysfunction has been shown to correlate with reduced radiosensitivity. We evaluated the levels and functional statuses of p53 in DLD-1, HCT8, HCT29, and HT116 (Figure 1C). In line with previous reports, p53 was found to be highly expressed in DLD-1 and HT29 cell lines (p53 has been mutated). However, contrary to what was reported previously, the radiation sensitivity of these CRC cell lines did not seem to correlate with their p53 functional status (Figure 1A vs. Figure 1C). Although p53 in DLD-1 has been mutated, this cell line belongs to the radiation-sensitive group, unlike HCT8, which expresses a WT p53 and belongs to the radiation-resistant group. There was no significant difference in 15-LOX-1 expression according to radiosensitivity in HCT8 and HCT116 cells, both of which are p53 WT. However, in p53 mutant cell lines, DLD-1 cells exhibited a high 15-LOX-1 expression and more radiation sensitivity than HT29 cells, which had low 15-LOX-1 expression. In other words, though radiosensitivity is not entirely determined by the state of p53 or the amount of 15-LOX-1 expression, it may be determined by the expression of 15-LOX-1 only in p53 mutant cell lines.

### 2.2. Radiation Induces Cell Death and Upregulates 15-Lox-1 Expression

**Figure 2:**
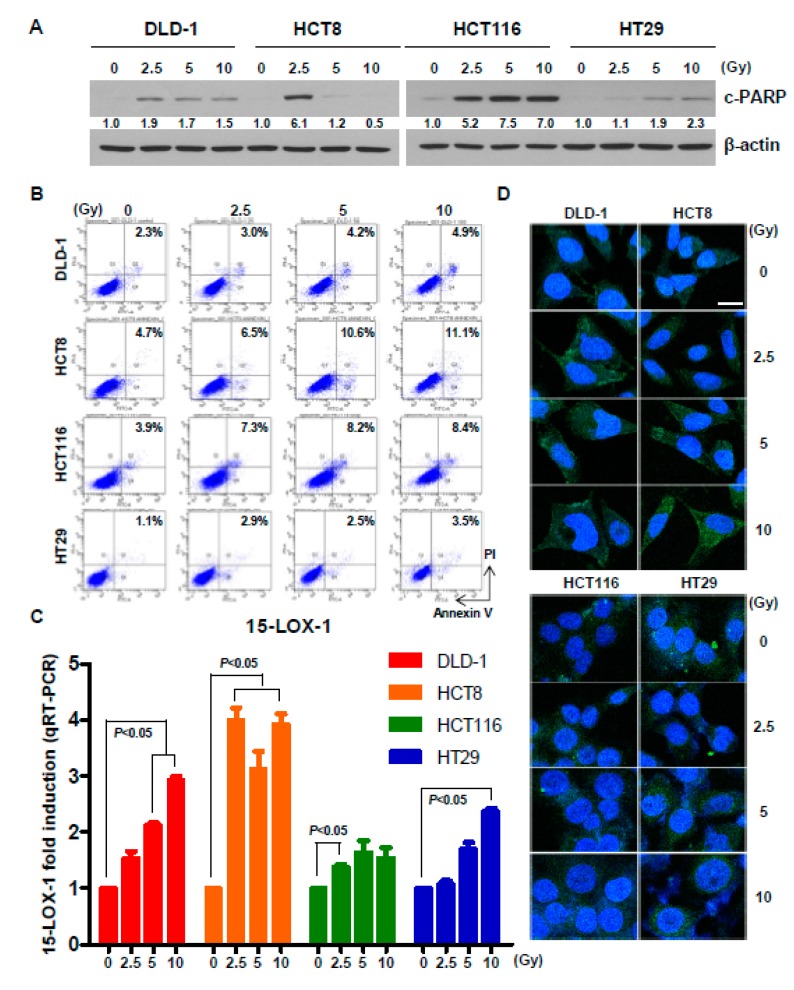
Radiation induces cell death and upregulates 15-LOX-1. (**A**) Twenty-four hours after irradiation at the indicated doses, cleaved PARP levels were measured by Western blotting, and (**B**) the number of Annexin V-positive cells increased, as demonstrated by flow cytometry. (**C**) Twenty-four hours after irradiation, the mRNA level of 15-LOX-1 in CRC cells was quantitated by qRT-PCR. (**D**) The 15-LOX-1 protein level was visualized by immunocytochemistry 24 h after irradiation. Scale bar, 10 μm.

To determine whether 15-LOX-1 expression is regulated by radiation, we irradiated CRC cell lines at 2.5, 5, or 10 Gy. First, we observed cell death upon irradiation. Twenty-four hours after irradiation, cleaved PARP levels (Figure 2A) and Annexin V-positive cell numbers (Figure 2B) were increased, as demonstrated by Western blotting and flow cytometry, respectively. Next, we determined the mRNA and protein levels of 15-LOX-1. Real-time PCR and immunocytochemistry (ICC) results showed that 24 h of irradiation significantly upregulated 15-LOX-1 in DLD-1 and HCT8 cells (Figure 2C,D). However, the 15-LOX-1 levels in HCT116 and HT29 cells were only slightly increased, especially at the protein level, as evidenced by the ICC results. Taken together, these results indicate that radiation induces 15-LOX-1 expression and causes cell death regardless of the p53 status. However, the degree of 15-LOX-1 induction was different in each cell line. A higher induction of 15-LOX-1 was observed in DLD-1 and HCT8, whose p53 states and radiation sensitivities did not match.

### 2.3. The Absence of 15-Lox-1 Decreases Radiation Sensitivity

**Figure 3:**
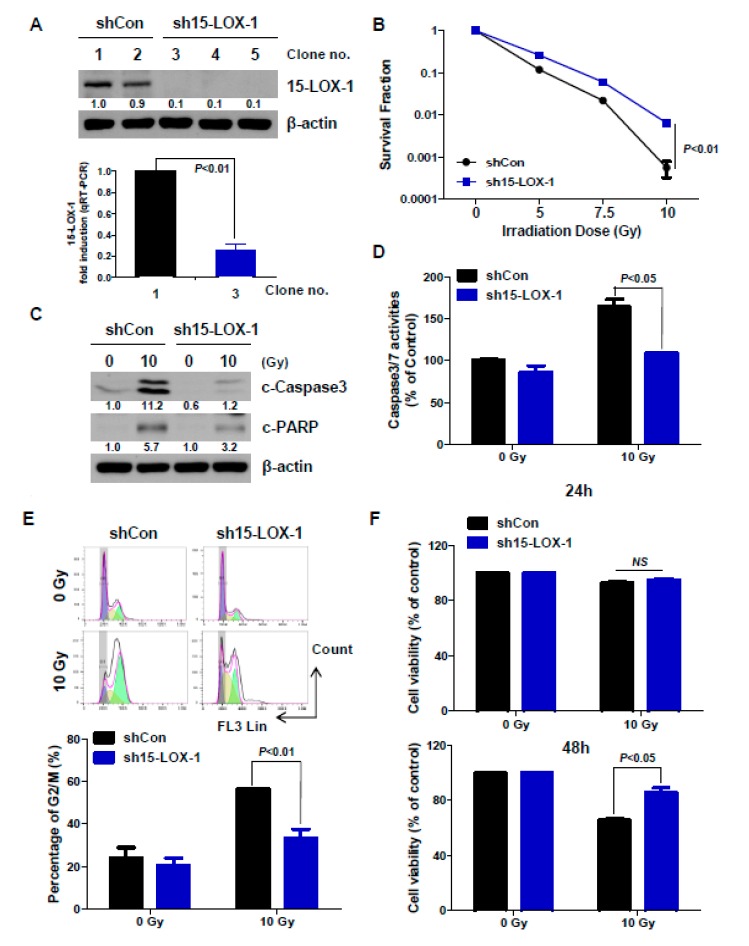
The absence of 15-LOX-1 decreased radiation sensitivity. (**A**) DLD-1 cells were transfected with a sh15-LOX-1 expression vector and clonally selected to obtain stable cell lines. The expression levels of 15-LOX-1 were double-checked, using Western blotting and qRT-PCR. (**B**) Radiosensitivity was measured through a clonogenic cell survival assay in both shCon and sh15-LOX-1 stable cell lines. (**C**) IR-induced apoptosis of the stable cell lines was analyzed by Western blotting 24 h after irradiation. (**D**) Caspase-3/7 activity in irradiated stable cells. (**E**) The G2/M percentage was determined by cell-cycle analysis. (**F**) Cell viabilities of the stable cell lines were evaluated, using the WTS assay, 24 and 48 h after irradiation.

To investigate the function of radiation-induced upregulation of 15-LOX-1, we generated stable cell lines, using a sh15-LOX-1 expression vector in DLD-1 cells (Figure 3A). The expression level of 15-LOX-1 in each clone was first measured by Western blotting; qRT-PCR was then conducted to confirm the 15-LOX-1 level in the selected clone (Clone no. 1: shCon stable cell line; Clone no. 3: sh15-LOX-1 stable cell line). The reason for selecting DLD-1 is that 15-LOX-1 is well induced by radiation, as shown in Figure 2C, and this cell line is a mutant for p53. We, thus, used DLD-1 cells to clarify the role of 15-LOX-1 in cells that are not affected by p53 signaling. Next, we investigated whether deficiencies in 15-LOX-1 expression are associated with a radiosensitive phenotype through a colony-forming assay for Clone No. 1 and No. 3 stable cells. Interestingly, sh15-LOX-1 cells were more resistant to IR than shCon cells (Figure 3B). We obtained the same results by using Western blotting, caspase-3/7 assay, and cell cycle analysis (Figure 3C–E). The conditions were the same 24 h after irradiation, and each experiment was conducted as described in Materials and Methods. Apoptosis, caspase-3/7 activation, and G2/M arrest occurred less in sh15-LOX-1 than in shCon stable cells. Lastly, cell viability was assessed 24 and 48 h after irradiation in these stable lines, using the WTS assay. The difference of cell viability that did not occur at 24 h after irradiation was significantly confirmed at 48 h after irradiation. Inhibition of cell viability by radiation occurred less in sh15-LOX-15 cells than in shCon cells. Together, these results indicate that lack of 15-LOX-1 decreases radiosensitivity in CRC cells.

### 2.4. MacroH2A2 is Transcriptionally Regulated by 15-Lox-1 in Crc Cells

**Figure 4:**
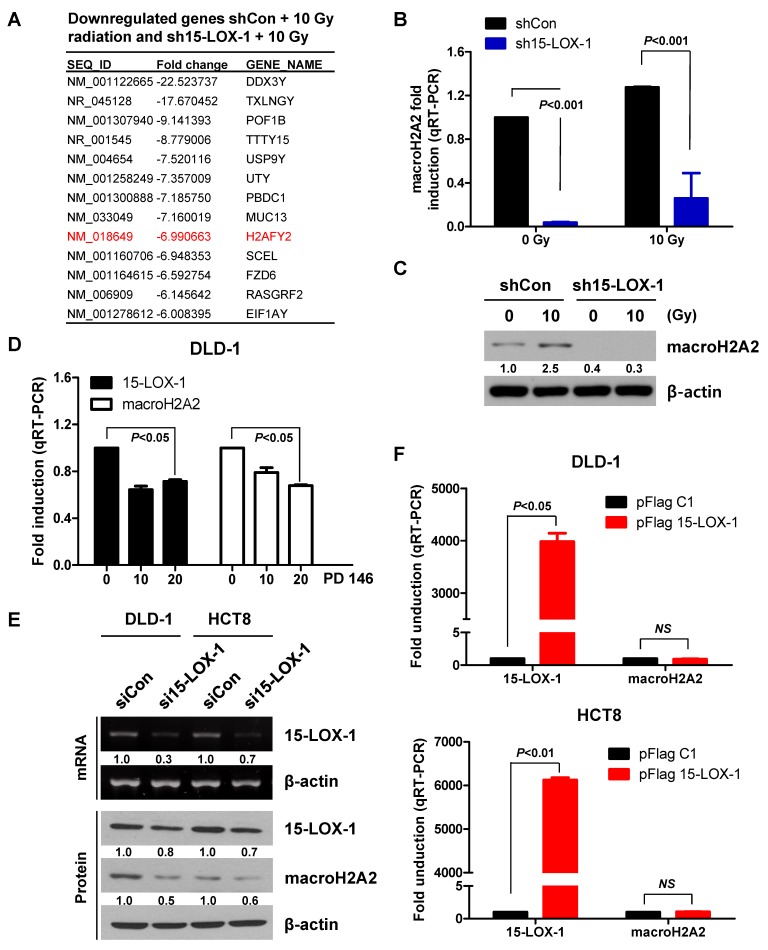
MacroH2A2 is transcriptionally regulated by 15-LOX-1 in colon cancer cells. (**A**) Genes downregulated by >sixfold between two groups. (**B**,**C**) MacroH2A2 levels of stable cell lines were measured by qRT-PCR and Western blotting 24 h after irradiation, to validate the microarray results. (**D**) MacroH2A2 expression in DLD-1 cells 24 h after PD146176 treatment. (**E**) MacroH2A2 expression in the transfected cells; mRNA levels of 15-LOX-1 were evaluated to confirm transfection efficiency. (**F**) MacroH2A2 mRNA levels were analyzed by qRT-PCR 24 h after transfection with a 15-LOX-1 vector.

To determine how 15-LOX-1 regulates radiosensitivity, we performed an Affymetrix expression microarray analysis of the stably transfected cell lines in triplicate. Each cell line was exposed to 10 Gy. We focused on the *H2AFY2* gene from the set of genes downregulated >sixfold (Figure 4A). We validated our microarray results by evaluating the macroH2A2 mRNA levels in the stable cell lines by qRT-PCR and protein levels by Western blotting (macroH2A2 is the protein encoded by the *H2AFY2* gene) (Figure 4B,C). The macroH2A2 level was found to be reduced in sh15-LOX-1 stable cells with or without radiation. The 15-LOX-1 inhibitor PD146176 downregulated macroH2A2 alongside 15-LOX-1, independent of irradiation (Figure 4D). We also assessed whether transient downregulation of 15-LOX-1 using RNAi could downregulate macroH2A2. We found that macroH2A2 RNA and protein levels decreased upon transient inhibition of 15-LOX-1 alone (Figure 4E). However, there was no change in the level of macroH2A2 mRNA when 15-LOX-1 was overexpressed through transfection of DLD-1 and HCT8 cells with the pFlag 15-LOX-1 vector (Figure 4F). These results indicate that macroH2A2 is transcriptionally regulated by 15-LOX-1 in CRC cells that significantly upregulate the 15-LOX-1 level upon irradiation.

### 2.5. Reduction in macroH2A2 Function Caused by 15-Lox-1 Transcriptional Downregulation Is Involved in Radioresistance through Suppression of the Radiation Response

**Figure 5:**
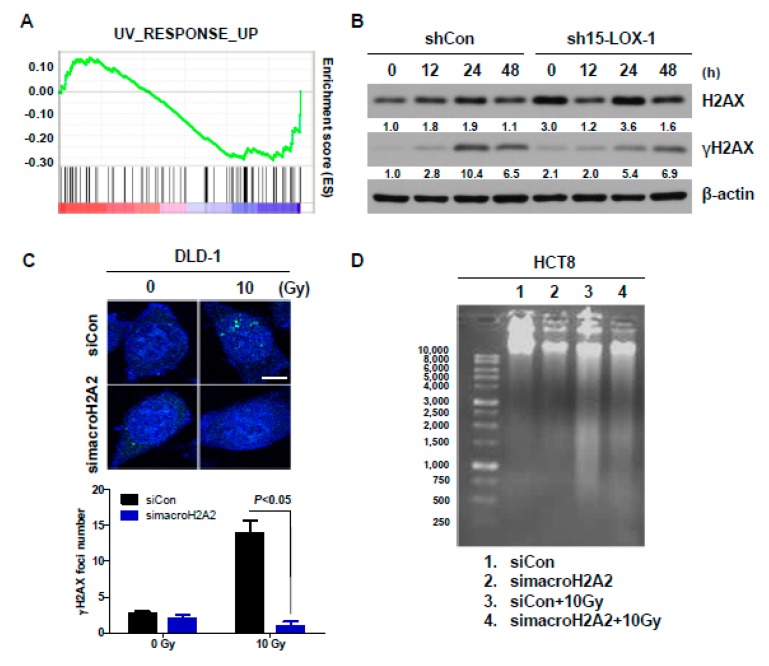

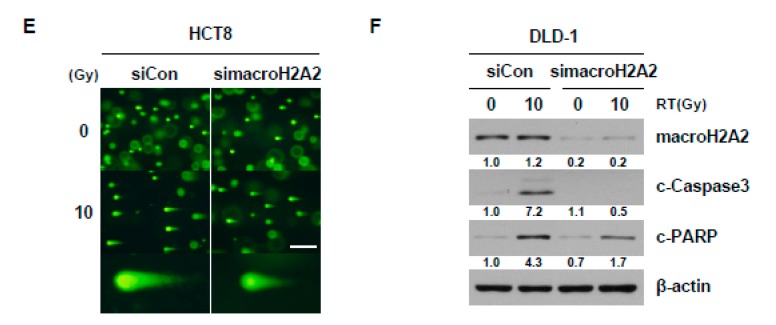
The reduction in macroH2A2 caused by 15-LOX-1 transcriptional downregulation is involved in radioresistance through reduction of the radiation response. (**A**) The downregulation of UV-RESPONSE_UP was observed by microarray data. (NOM *p*-value < 0.001, FDR *q*-value: 0.29, NES: −1.22) (**B**) Increased γH2AX expression in stable cell lines with time. (**C**) We incubated DLD-1 cells for 24 h after simacroH2A2 transfection. The transfected cells were irradiated at 10 Gy, and the formation of γH2AX foci was analyzed 24 h after irradiation. Scale bar, 10 μm. (**D**) A DNA fragmentation assay was conducted after transfection with simacroH2A2. (**E**) Comet assay results were visualized by using epifluorescence microscopy 24 h after irradiation. Scale bar, 200 μm. (**F**) Attenuation of IR-induced apoptotic cell death by simacroH2A2 in DLD-1 cells.

Although the function of macroH2A2 in response to DNA damage has not been clearly identified yet, we searched for a connection between macroH2A2 function and radiation response, using gene set enrichment analysis (GSEA) of the microarray data. GSEA was performed on expression data, including those regarding 706 genes, using the conditions FDR < 0.01 and |FC| ≥ 0.5. The analysis showed that the UV response was decreased in sh15-LOX-1 stable cells (Figure 5A). Therefore, we assessed the levels of the DNA DSB marker phosphorylated H2AX (Ser139, γH2AX) during the indicated periods in the stable cell lines. H2AX was activated by 10 Gy after 24 h in shCon, but not sh15-LOX-1 cells (Figure 5B). It appears that the reduction in 15-LOX-1 activity was associated with a decreased DNA damage response. To investigate the relationship between DNA damage response and 15-LOX-1–regulated macroH2A2 function, DLD-1 cells were transfected with siRNA against macroH2A2 (simacroH2A2) and exposed to 10 Gy 24 h after the transfection. The generation of γH2AX foci by radiation was decreased 24 h after irradiation in simacroH2A2-transfected cells (Figure 5C). To further determine the extent of DNA damage relative to the expression level of macroH2A2, cells were transfected with simacroH2A2 and exposed to radiation 24 h after transfection. The DNA damage of the cells was evaluated by DNA fragmentation (Figure 5D) and comet assays (Figure 5E) 24 h after irradiation. Radiation-induced DNA damage was found to be attenuated upon silencing of macroH2A2 (Figure 5D,E). In addition, we assessed apoptotic cell death markers by Western blotting. Compared with the DLD-1 cells transfected with siCon, the decrease in macroH2A2 activity in siRNA-treated cells attenuated IR-induced apoptotic cell death (Figure 5F). The results indicated that reduced macroH2A2 level upon 15-LOX-1 downregulation increases radiation resistance by suppressing the radiation response.

## 3. Discussion

Although the relationship between p53 and the radiation response is well-known [25,26,27,28], p53 is not the only factor that controls the radiosensitivity of cells. Previous studies have suggested that 15-LOX-2 and 12-LO are also involved in regulating radiosensitivity both in head and neck and prostate cancers [22,23,24]. Even though radiation therapy is essential for the timely treatment of CRC to prevent anus amputation, few biomarkers are available to predict CRC patient response to radiation therapy. We hypothesized that 15-LOX-1 could be a regulator of radiosensitivity. In our clonogenic cell survival assay, radiosensitivity was found to be correlated with the expression level of 15-LOX-1 protein and *p*53 mutational status (Figure 1).

Histones are commonly known as nuclear proteins that are involved in DNA repair, DNA replication, and chromosomal stability. MacroH2A2 is one of the several variants of histone H2A. Until this study, macroH2A2 was known to function only in X inactivation [29,30]. This study offers the first demonstration of a relationship between macroH2A2 and the DNA damage response. Although we do not yet understand in detail how macroH2A2 modulates the DNA damage response, γH2AX regulation of macroH2A2 was identified as a novel mechanism (Figure 5). To better understand the specificity of this regulation, we plan to evaluate the expression levels of DNA DSB sensors, members of the MRN complex (MRE11, Rad50, and NBS1), and ATM in cells deficient for macroH2A2. We will also confirm the binding of these genes because the γH2AX signal does not appear until ATM binds to the MRN complex.

If 15-LOX-1 primarily functioned as a transcription factor, the mechanism by which it regulates macroH2A2 would be more obvious. However, most 15-LOX-1 is found in the cytoplasm and not in the nucleus. Several transcription factors, such as c-Myb, Oct-1, and Oct-2, are known to regulate macroH2A2. We hypothesized that 15-LOX-1 regulates macroH2A2 transcription via these promoter-binding proteins. The protein levels of Oct-1 and Oct-2 were found to decrease in sh15-LOX-1 stable cells, si15-LOX-1 transient cells, and PD146176-treated cells (data not shown). In addition, Oct-2 transcription is regulated by binding to the Oct-2 promoter of PPAR-gamma activated by 15-LOX-1. How these genes correlate with each other will be assessed in the future.

Additionally, the gene set enrichment analysis (GSEA) also suggested that the markers of the epithelial-to-mesenchymal transition (EMT) were significantly upregulated in sh15-LOX-1 stable cells. Especially N-cadherin, which is a hallmark of EMT, was upregulated in sh15-LOX-1 stable cells irrespective of irradiation (Supplementary Figure S1). When these cells were exposed to radiation, their N-cadherin level seemed to decrease slightly but remained higher than that in shCon stable cells. Radiation-induced EMT is one of the mechanisms underlying resistance to radiation [31,32]. Therefore, it will be another interesting topic in the future.

Patient-specific treatment is becoming more common for the elimination of unnecessary chemotherapy, but it is used less often for radiotherapy. The discovery and characterization of biomarkers that can predict the efficacy of radiation therapy has the potential to improve cancer treatment. This study suggests that 15-LOX-1 has significant potential as a biomarker indicating the radiosensitivity of CRC cells.

## 4. Materials and Methods

### 4.1. Cell Culture

The human CRC cell lines DLD-1, HCT8, HT29, and HCT116 were obtained from the American Type Culture Collection (ATCC; Manassas, VA, USA). Cells were grown in RPMI 1640 and McCoy’s 5A medium (Invitrogen, Carlsbad, CA, USA) supplemented with 10% fetal bovine serum (FBS) and L-glutamine, in a humidified chamber, at 37 °C, and under 5% CO_2_.

### 4.2. Reagents and Antibodies

The 15-LOX-1 inhibitor PD 146176 was purchased from Selleckchem (Houston, TX, USA) and Calbiochem (San Diego, CA, USA). Anti-15-LOX-1 was purchased from Abcam (Cambridge, MA, USA). Anti-p53, anti-macroH2A2, and FITC-conjugated anti-15-LOX-1 were purchased from Santa Cruz Biotechnology (Santa Cruz, CA, USA). Anti-cleaved PARP, anti-cleaved Caspase3, anti-H2AX, and anti-γH2AX were obtained from Cell Signaling (Beverly, MA, USA). Anti-actin was purchased from Sigma (St. Louis, MO, USA).

### 4.3. Western Blot Analysis

Proteins were extracted, using RIPA buffer supplemented with protease and phosphatase inhibitors (Sigma-Aldrich, St. Louis, MO, USA). Equal amounts of proteins were analyzed by SDS PAGE after quantitation through the bicinchoninic acid (BCA, Thermo Fisher Scientific, Hudson, NH, USA) protein assay. Membranes with the transferred proteins were blocked with skim milk and sequentially incubated with primary and secondary antibodies. Chemiluminescent reagents (DoGen, Seoul, Korea) were used to visualize protein levels on X-ray films. Detailed information of western blot can be found at Supplementary Figure S2.

### 4.4. Polymerase Chain Reaction (PCR)

The cDNA synthesis was conducted by using the reverse transcriptase PCR kit (Life Technologies, Gaithersburg, MD, USA) and RNA extracted from CRC cell lines. Real-time PCR was carried out via Taqman GAPDH (Hs99999905_m1), macroH2A2 (Hs01013229_m1), and 15-LOX-1 (Hs00993765_ g1) probes (Applied Biosystems). GAPDH mRNA levels were used for normalization of target gene mRNA levels. PCR amplification of 15-LOX-1 was performed, using the following primers:

Forward: 5′-GCTGCGGCTCTGGGAAATCATCT-3′.

Reverse: 5′-GGGCCCGAAAAATACTCCTCCTCA-3′.

### 4.5. Clonogenic Cell Survival Assay

CRC cells were counted and plated (5 × 10^2^ cells per 60 mm dish) in triplicate and exposed to various doses of radiation the next day. Depending on their clonogenic ability, cells were grown for 14–18 days. Cells were finally washed and stained with crystal violet. Colonies containing >50 cells were counted. Specifically, 137Cs-γ-rays (Atomic Energy of Canada) were used for in vitro experiments, at a dose rate of 3.81 Gy/min.

### 4.6. Microarray Analysis

Stable cells were irradiated with 10 Gy radiation. After 6 h, RNA was extracted, using the TRIzol reagent. Reverse transcription was carried out, and the Affymetrix Whole Transcript Expression array was used, according to the manufacturer’s instructions, for microarray analysis (GeneChip Whole Transcript PLUS reagent kit and GeneChip Whole Transcript Amplification kit). The cDNA labeling (GeneChip WT Terminal labeling kit), hybridization (Affymetrix GeneChip 2.0 ST Array), and slide scanning (Affymetrix, GCS3000 Scanner) were performed according to the manufacturers’ instructions.

### 4.7. Transfection

CRC cells were transfected with 15-LOX-1 siRNA (sc-105003) and macroH2A2 siRNA (sc-62575) (Santa Cruz Biotechnology), using Lipofectamine RNAiMax (Invitrogen), and with the 15-LOX-1 shRNA Expression Vector (GenePharma, Shanghai, China) and pFlag 15-LOX-1 vector, using Lipofectamine 2000 (Invitrogen), according to the manufacturer’s instructions.

### 4.8. Cell-Cycle Assay

Cells were harvested, washed twice with PBS, and fixed in 85% ethanol containing 5 mM EDTA. The fixed cells were treated with 20 µg/mL RNaseA, followed by staining with 50 µg/mL propidium iodide (PI, Sigma-Aldrich). DNA content was determined through the use of a Beckman Coulter flow cytometer. Results were processed by using the FlowJo program for the determination of cell numbers in the G1, S, and G2/M phases.

### 4.9. Analysis of 15-LOX-1 Using Flow Cytometry

Cultured cells were trypsinized and harvested in a conical tube. Subsequently, they were fixed in 3.7% paraformaldehyde for 15 min and permeabilized with 90% methanol for 5 min, at 25 °C. Cells were incubated with FITC-conjugated anti-15-LOX-1 (sc-133085 FITC) for 30 min, in an ice bucket protected from light. Unstained cells were used as a negative control.

### 4.10. Immunofluorescent Staining

Cells plated on glass coverslips were exposed to radiation; then, they were fixed and permeabilized with 3.7% paraformaldehyde and 0.5% Triton X-100, respectively, for 15 min each, at 25 °C, and blocked with 3% bovine serum albumin for 30 min, in a covered ice bucket, the following day. After blocking, cells were incubated with FITC-conjugated anti-15-LOX-1 and anti-γH2AX, overnight, at 4 °C. Lastly, cells were washed and stained with Alexa Fluor 488-labeled secondary antibody (only for γH2AX) and DAPI, in consecutive order.

### 4.11. Caspase 3/7 Assay

Cells were seeded in 96-well plates. On the next day, cells were treated with 100 µL Caspase-Glo^®^ 3/7 Reagent (Promega, Madison, WI, USA) for 0.5–3 h at 25 °C. Then, caspase-3/7 activity was quantitated with a luminometer. Untreated cells were used as a negative control.

### 4.12. Annexin V Assay

Collected cells were resuspended in the binding buffer and stained with 1.25 μL of Annexin V-FITC and 10 μL of propidium iodide (PI) for 30 min, at 4 °C (ApoScan Annexin V-FITC apoptosis detection Kit, BioBud, Seoul, Korea). The results were analyzed with a BD flow cytometer.

### 4.13. Cell Viability Assay

Cells were grown in 96-well plates and exposed to 10 Gy radiation. To confirm the effect of radiation, cells were incubated for 24 or 48 h. Cell viability was measured by using the EZ-Cytox kit reagent (DoGen, Seoul, Korea). Absorbance at 450 nm was used to analyze cell viability.

### 4.14. Apoptotic DNA Ladder Detection

Cells were lysed with 35 μL of TE Lysis Buffer and then incubated with 5 μL each of Enzyme A and Enzyme B, to digest RNA and protein, respectively. Next, the DNA of the cells was precipitated, using 5 μL of ammonium acetate. The DNA pellet was resuspended in the DNA suspension buffer and analyzed by 1.2% agarose gel electrophoresis. The results were visualized through the use of a transilluminator.

### 4.15. Comet Assay

Cells (1 × 10^5^ cells/mL) were mixed with Comet Agarose at a 1:10 ratio and plated onto a Comet Slide. The slide was then incubated at 4 °C, for 15 min, to harden the agarose. Then, it was sequentially immersed in prechilled lysis buffer and alkaline solution for 30 min, at 4 °C. The Comet Agarose gel was stained with Vista Green DNA Dye after electrophoresis (OxiSelect Comet Assay Kit, Cell Biolabs, Inc., San Diego, CA, USA).

### 4.16. Statistical Analysis

Statistical analyses were carried out by using the Prism5 software (GraphPad Software, Inc., San Diego, CA, USA). Results are expressed as the mean ± standard error of the mean (SEM). All the results were evaluated by using unpaired Student’s *t*-test, and a *p*-value < 0.05 was considered significant.

## 5. Conclusions

In this paper, 15-LOX-1 is proposed as a biomarker for the radiosensitivity of which cannot be determined based on the *p53* status.

## Supplementary Materials

The following are available online at https://www.mdpi.com/2072-6694/11/11/1776/s1, Figure S1: The markers of the epithelial-to-mesenchymal transition (EMT), especially N-cadherin, are significantly upregulated in sh15-LOX-1 stable cells, Figure S2: Immunoblots with molecular weight markers.
