# Supplementary material for: Deficiency of 15-LOX-1 Induces Radioresistance through Downregulation of MacroH2A2 in Colorectal Cancer
Source: Cancers (Basel). 2019 Nov 11;11(11):1776. doi: 10.3390/cancers11111776 (PMC6896202; doi:10.3390/cancers11111776)
Supplement: Supplementary file 1 [file cancers-11-01776-s001.pdf]

Supplementary Materials

Deficiency of 15-LOX-1 Induces Radioresistance through Downregulation of MacroH2A2 in Colorectal Cancer

Yoo Jin Na, Bo Ram Kim, Jung Lim Kim, Sanghee Kang, Yoon A Jeong, Seong Hye Park, Min Jee Jo, Jeong-Yub Kim, Hong Jun Kim, Sang Cheul Oh and Dae-Hee Lee

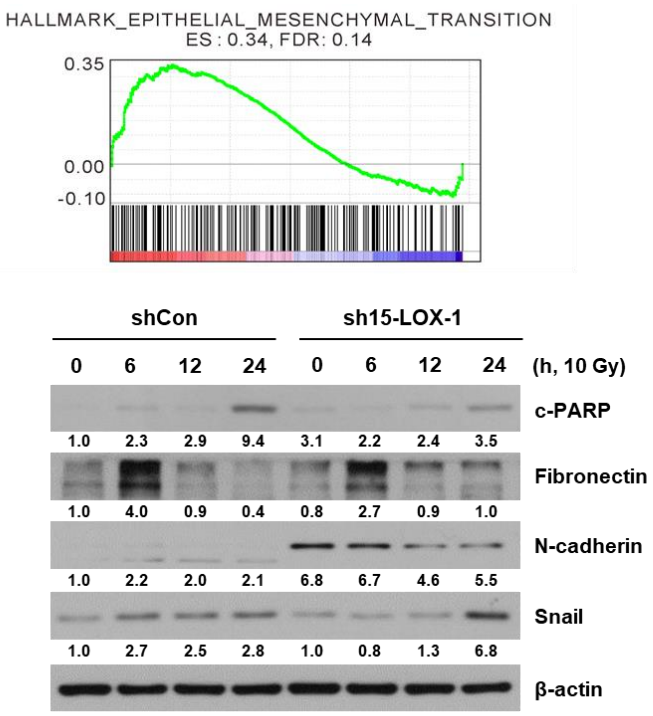

Figure S1. The markers of the epithelial-to-mesenchymal transition (EMT), especially N-cadherin, are significantly upregulated in sh15-LOX-1 stable cells.

Figure 1C

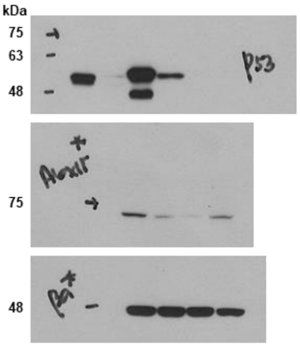

Figure 2A

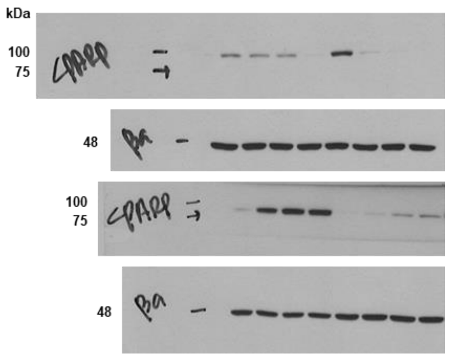

Figure 3A

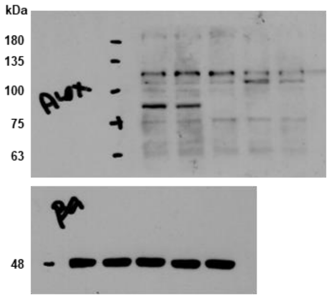

Figure 3C

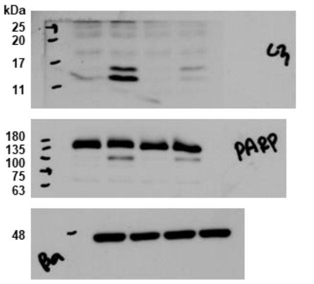

Figure 4C

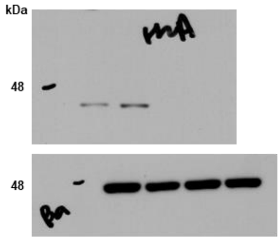

Figure 4E

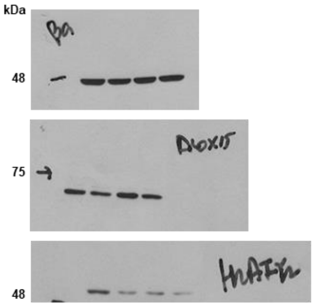

Figure 5B

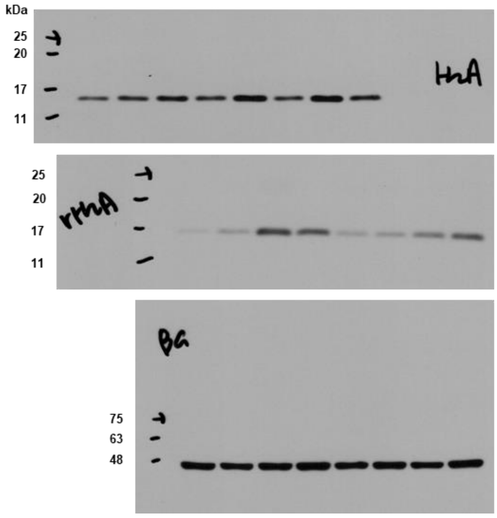

Figure 5F

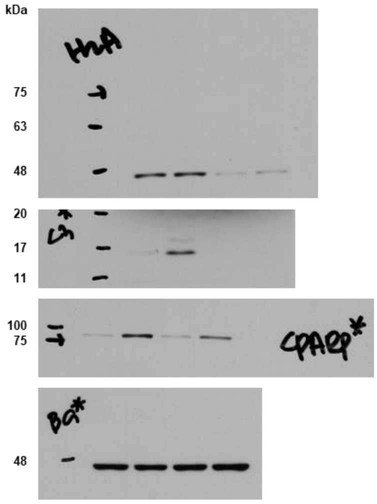

Supplementary Figure S1

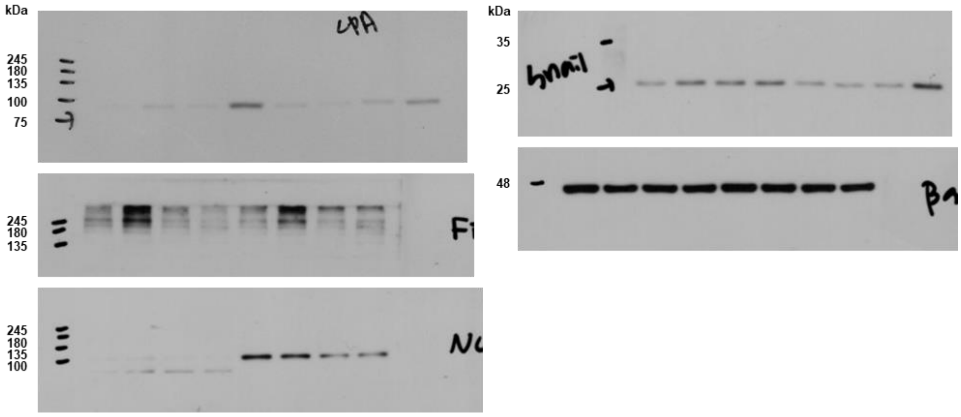

Figure S2. Immunoblots with molecular weight markers.

© 2019 by the authors. Licensee MDPI, Basel, Switzerland. This article is an open access article distributed under the terms and conditions of the Creative Commons Attribution (CC BY) license (http://creativecommons.org/licenses/by/4.0/).
